# Key Enzymes in Pyrimidine Synthesis, CAD and CPS1, Predict Prognosis in Hepatocellular Carcinoma

**DOI:** 10.3390/cancers13040744

**Published:** 2021-02-11

**Authors:** Dirk Andreas Ridder, Mario Schindeldecker, Arndt Weinmann, Kristina Berndt, Lana Urbansky, Hagen Roland Witzel, Stefan Heinrich, Wilfried Roth, Beate Katharina Straub

**Affiliations:** 1 Institute of Pathology, University Medical Center of the Johannes Gutenberg University, 55131 Mainz, Germany; Mario.Schindeldecker@unimedizin-mainz.de (M.S.); k.berndt@students.uni-mainz.de (K.B.); lana.urbansky@t-online.de (L.U.); hagen.witzel@unimedizin-mainz.de (H.R.W.); wilfried.roth@unimedizin-mainz.de (W.R.); 2 Tissue Biobank, University Medical Center of the Johannes Gutenberg University, 55131 Mainz, Germany; 3 Department of Internal Medicine, University Medical Center of the Johannes Gutenberg University, 55131 Mainz, Germany; arndt.weinmann@unimedizin-mainz.de; 4 Department of General, Visceral and Transplant Surgery, University Medical Center of the Johannes Gutenberg University, 55131 Mainz, Germany; Stefan.Heinrich@unimedizin-mainz.de

**Keywords:** hepatocellular carcinoma, HCC, prognosis, biomarker, pyrimidine, cps1, cad, urea cycle dysregulation

## Abstract

**Simple Summary:**

Individual patients with liver cancer have a highly variable clinical course. Hence, there is an urgent need to identify new prognostic markers to determine prognosis and select specific therapies. Expression of two key enzymes in pyrimidine synthesis was analyzed in a large, well-characterized cohort of patients with liver cancer. Dysregulated expression of these enzymes was associated with shorter survival of the patients. A combined score of both markers was found to be a statistically independent prognostic marker.

**Abstract:**

Patients with hepatocellular carcinoma (HCC) have a highly variable clinical course. Therefore, there is an urgent need to identify new prognostic markers to determine prognosis and select specific therapies. Recently, it has been demonstrated that dysregulation of the urea cycle (UC) is a common phenomenon in multiple types of cancer. Upon UC dysregulation, nitrogen is diverted toward the multifunctional enzyme carbamoyl-phosphate synthetase 2, aspartate transcarbamoylase, and dihydroorotase (CAD), and increases pyrimidine synthesis. In this study, we investigated the role of CAD and carbamoyl-phosphate synthetase 1 (CPS1), a rate-limiting enzyme of the UC highly expressed in hepatocytes, in HCC. We created a tissue microarray to analyze expression of both enzymes by immunohistochemistry in a large and well-characterized overall cohort of 871 HCCs of 561 patients that underwent surgery. CAD was induced in recurrent HCCs, and high expression predicted shorter overall survival. CPS1 was downregulated in HCC and further reduced in recurrent tumors and distant metastases. Additionally, low CPS1 was associated with short overall survival. A combined score of both enzymes was an independent prognostic marker in a multivariate Cox regression model (HR = 1.37, 95% confidence interval 1.06–1.75, *p* = 0.014). Inhibition of pyrimidine synthesis may represent a novel therapeutic strategy for HCC.

## 1. Introduction

Primary liver cancer is the fourth most common cause of cancer-related death worldwide, with about 782,000 deaths in 2018 [1]. Hepatocellular carcinoma (HCC) accounts for the vast majority of cases of primary liver cancer. Clinical prognosis assessment and decision processes are currently based on one of the several tumor staging systems (for example, the Barcelona Clinic Liver Cancer (BCLC), Japan Integrated Staging (JIS), or Hong-Kong Liver Cancer (HKLC) staging system). Although these staging systems allow quite robust stratification in different prognostic groups, the clinical course of individual patients suffering from HCC is highly variable, and there is still room for refinement in the evaluation of prognosis, especially in early tumor stages [2]. For that reason, more accurate prognostic markers are needed to determine prognosis and select specific treatment options. Furthermore, it is important to further elucidate the molecular mechanisms underlying the development and progression of HCC in order to develop new therapies and improve survival rates.

Generally, in cancer cells, metabolism is reprogrammed in favor of maximal anabolic synthesis of macromolecules needed to maintain viability and proliferation [3]. In hepatocytes, the urea cycle (UC) serves to convert excess nitrogen derived from nitrogen-containing compounds, such as glutamine and ammonia, into disposable urea [4]. In other organs, different components of the UC are differentially expressed to meet the specific local needs for urea cycle intermediates. Besides an increased consumption of carbon, cancer cells also have an increased demand for reduced nitrogen [3,5]. Recently, it has been demonstrated that UC dysregulation is a common phenomenon in multiple types of cancer [6].

Upon UC dysregulation, nitrogen is redirected toward the multifunctional enzyme carbamoyl-phosphate synthetase 2, aspartate transcarbamoylase, and dihydroorotase (CAD), and increases pyrimidine synthesis [6,7]. CAD is a single polypeptide with three distinct functional domains, with the carbamoyl-phosphate synthetase domain catalyzing the rate-limiting step of pyrimidine synthesis [8]. CAD expression has been shown to be tightly linked to proliferation; overexpression increases, and knock-down decreases, proliferation rates [7,9,10]. It is predominantly localized in the cytoplasm, and has been shown to be induced in glioblastoma and to predict recurrence in prostate adenocarcinoma [7,11], but its role in hepatocellular carcinoma has not been investigated so far.

The mitochondrial counterpart of CAD, carbamoyl-phosphate synthetase 1 (CPS1), catalyzes the initial and rate-limiting step of the urea cycle by generating carbamoyl phosphate from NH3 and CO2 [5]. CPS1 was discovered as the target of the hepatocyte paraffin 1 (HepPar1) antibody [12], which is most often used by pathologists to differentiate hepatocellular carcinoma from metastases, as it is highly expressed in cells of hepatocellular origin, whereas other organs only show low expression levels [13]. CPS1 has been demonstrated to be downregulated in hepatocellular carcinoma by hypermethylation of the CPS1 promoter [14,15], whereas CAD mRNA levels were increased in HCC tissue [15]. Macrovascular invasion, one of the most significant predictors of early HCC recurrence, was associated with a downregulation of different UC enzymes, including CPS1 [16]. A reduction in CPS1 in HCC may result in increased shunting of glutamine to CAD, which is the initiating step of the de novo pyrimidine synthesis pathway, and may lead to unfavorable outcomes [17]. In order to elucidate the role of CAD and CPS1 and their effect on prognosis in HCC, we investigated the expression of both enzymes in a large collective of HCCs in comparison to respective non-neoplastic liver tissue by immunohistochemistry and correlated their expression with clinical and histopathological features.

## 2. Results

### 2.1. CAD Is Induced in Recurrent Hepatocellular Carcinoma and Predicts Prognosis

**Figure 1:**
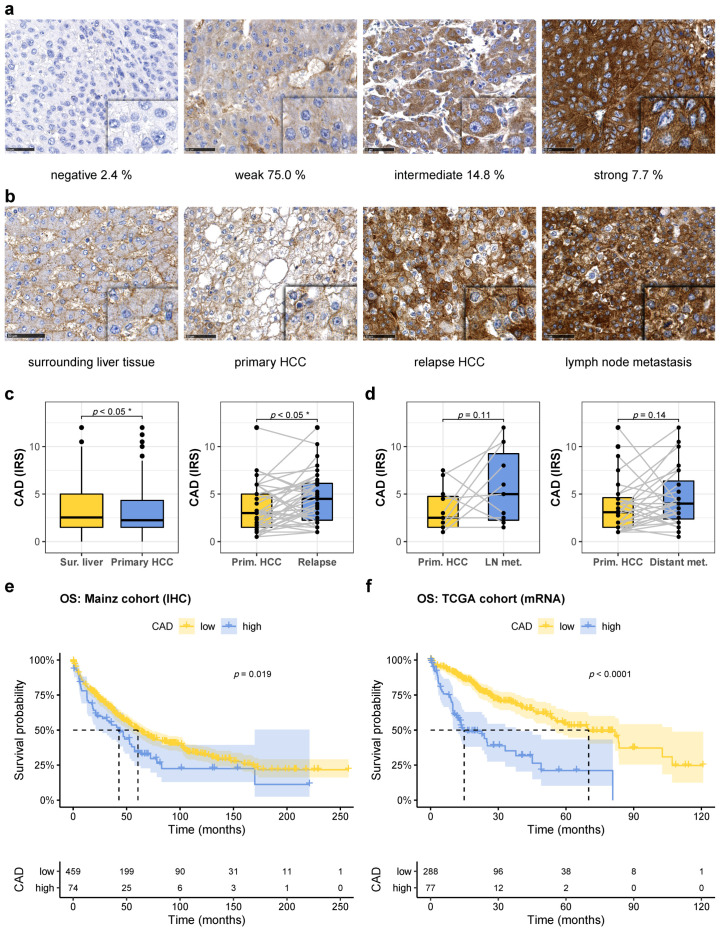
CAD is induced in recurrent hepatocellular carcinoma and predicts prognosis. (**a**) Representative images of immunohistochemical staining of primary HCCs with different expression levels of CAD. The percentage of tumors with the indicated expression level is shown. Scale bar: 50 µm. (**b**) Increased expression of CAD during tumor progression. Immunostainings for CAD in surrounding liver tissue, primary and relapse HCC, and lymph node metastasis of one individual patient. Scale bar: 50 µm. (**c**) Quantitative analysis of CAD expression (immunoreactive score, IRS) in primary HCC compared to non-neoplastic surrounding tissue (left panel, surrounding liver: *n* = 530, HCC: *n* = 533) and in recurrent HCC compared to primary HCC (right panel, *n* = 39); * *p* ≤ 0.05. (**d**) Quantitative analysis of CAD expression in primary HCC compared to lymph node metastasis (left panel, *n* = 11) and distant metastasis (right panel, *n* = 28). (**e**) Kaplan–Meyer plot displaying overall survival with respect to high and low CAD protein expression as detected by immunohistochemistry (HR 1.44, 95% confidence interval 1.06–1.95, *p* = 0.019). (**f**) Kaplan–Meyer plot displaying overall survival with respect to high and low expression of CAD mRNA in the TCGA cohort (HR 3.31, 95% confidence interval 2.28–4.82, *p* ≤ 0.0001).

To test whether the rate-limiting enzyme of the pyrimidine pathway, CAD, is of clinical and prognostic relevance, and to be able to investigate CAD expression in a large number of HCCs, we established a tissue microarray (TMA) of 871 HCCs of overall 561 patients that underwent surgery or liver transplantation with comprehensive clinicopathologic and clinical data (see Table S1 for summary of clinical data). We detected CAD protein in HCC and surrounding tissue at varying amounts and at high levels in cultured HepG2 and Huh7 cells by immunoblotting at the predicted molecular weight of around 250 kDa (Figures S1a and S5). Expression levels detected by immunohistochemistry employing the same antibody correlated well with detection by immunoblot (Figures S1a,c and S5). For semiquantitative assessment of CAD expression, we employed the immunoreactive score (IRS) [18]. Of the HCCs, 2.4% showed no detectable CAD expression (IRS = 0), whereas 75.0% displayed weak (IRS = 1–4), 14.8% intermediate (IRS = 5–8), and 7.7% strong (IRS = 9–12) CAD staining, which was predominantly localized in the cytoplasm (Figure 1a and Figure S1b,c). While CAD has been demonstrated to be induced on the mRNA level in HCC tissue in the Cancer Genome Atlas (TCGA) cohort [15], increased mRNA levels did not translate into an increase in CAD protein levels in primary HCCs. In fact, we detected a slight but significant downregulation of CAD in primary HCCs on the protein level when compared to the surrounding non-neoplastic liver tissue (Figure 1b,c, left panel), but recurrent HCCs presented with significantly increased CAD levels when compared to the primary HCCs (Figure 1b,c, right panel). Whether recurrent tumors constitute true recurrences or de novo HCCs is difficult to determine [19]. According to the literature, the majority of tumors that reoccur within the first two years after resection or transplantation are considered true recurrences and are not de novo HCCs [20,21,22,23]. When we restricted expression analysis to those early, probably true recurrences, CAD expression was still significantly induced when compared to the primary HCC (Figure S2a). Furthermore, in lymph node and distant metastases, a trend towards increased CAD levels was detected when compared to the respective primary tumors (Figure 1b,d), which did not reach significance, possibly due to the small numbers of available samples of HCC metastases. High CAD protein expression in primary HCCs (IRS > 6.125) was associated with reduced overall survival (OS) rates (Figure 1e). This finding was further confirmed on the mRNA level in the publicly available, independent TCGA cohort of HCC patients (Figure 1f).

In order to further elucidate regulation of CAD expression in HCC, we analyzed the TCGA dataset for correlation of CAD mRNA with known transcriptional regulators of CAD, which comprise MYC [24] and estrogen receptor α (ESR1) [25]. CAD expression in the TCGA dataset inversely correlated with ESR1 (R = −0.38, *p* = 6.62 × 10^−14^) and correlated with MYC (R = 0.27, *p* = 1.18 × 10^−7^). These two factors may contribute to the regulation of CAD expression in HCC. Furthermore, we found CAD mRNA levels to moderately correlate with DNA methylation of the CAD locus (Spearman R = 0.48, *p* = 3.45 × 10^−22^).

### 2.2. High CAD Expression Is Associated with Unfavorable Prognostic Factors

**Figure 2:**
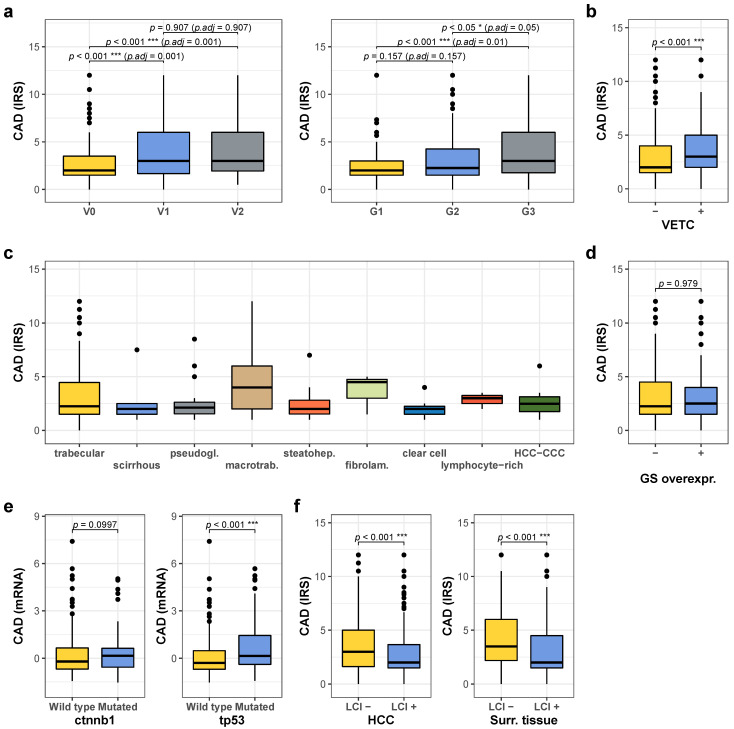
Comparison of CAD expression with clinicopathological and molecular parameters. (**a**) Quantification of CAD expression detected by immunohistochemistry with respect to vascular invasion (left panel, V0: *n* = 344, V1: *n* = 109, V2: *n* = 80) and tumor grade (right panel, G1: *n* = 99, G2: *n* = 265, G3: *n* = 115). (**b**) Quantification of CAD expression in relation to absence or presence of the VETC pattern (VETC-: *n* = 438, VETC+: *n* = 95). (**c**) Quantification of CAD expression according to morphological tumor subtype (patient numbers: trabecular: 418, scirrhous: 5, pseudogl.: 18, macrotrab.: 41, steatohep.: 22, fibrolam.: 3, clear cell: 7, lymphocyte-rich: 3, HCC-CCC: 8). (**d**) CAD expression levels in relation to absence or presence of GS overexpression as a marker for CTNNB1 mutations/activated WNT-signaling (GS neg.: *n* = 423, pos.: *n* = 109). (**e**) Quantification of CAD mRNA in the TCGA cohort with respect to mutational status of CTNNB1 (left panel, wild type: *n* = 264, mutated: *n* = 96) and TP53 (right panel, wildtype: *n* = 249, mutated 111). (**f**) Quantification of CAD expression in tumor tissue (left panel, no cirrhosis: *n* = 192, cirrhosis: *n* = 341) and in the surrounding liver tissue (right panel, no cirrhosis: *n* = 188, cirrhosis: *n* = 342) with respect to absence or presence of liver cirrhosis. For all analyses * denotes *p* ≤ 0.05, *** p ≤ 0.001.

CAD expression was then correlated with clinicopathologic and molecular parameters. Tumors with high CAD levels occurred significantly more often in females and less often in association with alcohol abuse (for clinicopathologic features with respect to low and high CAD expression, see Table 1). Tumors with high CAD expression presented with a disproportionately high rate of portal vein thrombosis detected by preoperative imaging, and of micro- and macrovascular invasion detected upon pathologic work-up of the resected tissue. In line with this finding, HCCs with micro- and macrovascular invasion displayed higher CAD levels than tumors without vascular invasion (Figure 2a, left panel). Furthermore, G3 tumors exhibited increased CAD levels (Figure 2a, right panel). Additionally, tumors with vessels encapsulating tumor clusters (VETC) pattern (as determined by CD34 immunohistochemistry), a morphological feature previously shown to be associated with vascular invasion and worse prognosis [26], exhibited significantly higher CAD levels (Figure 2b).

Recently, different histological subtypes of HCCs have been proposed with characteristic morphologies and distinct prognoses [27]. Presence of the macrotrabecular-massive subtype has particularly been linked to worse clinical outcome [28]. In line with this, we detected significantly higher CAD levels in tumors with macrotrabecular-massive growth pattern (Figure 2c). CAD expression was not significantly associated with glutamine synthetase (GS) overexpression as an immunohistochemical surrogate parameter of WNT activation in, for example, CTNNB1-mutated HCC [29] (Figure 2d) or with CTNNB1 mutations in the TCGA cohort (Figure 2e, left panel), whereas TP53-mutated tumors exhibited significantly increased CAD expression (Figure 2e, right panel). Interestingly, liver cirrhosis was associated with significantly lower CAD levels in HCC and surrounding tissue (Figure 2f). Furthermore, high CAD expression levels in tumor tissue were associated with obesity (BMI > 30) (Figure S2c) and lower CAD levels with alcohol abuse (Figure S2d), whereas no significant differences were detected with respect to HBV or HCV infection or hemochromatosis (not shown).

In the surrounding non-neoplastic liver tissue, CAD was increased in patients suffering from HBV infection (Figure S2e) and decreased upon HCV infection (Figure S2g), whereas no differences were observed depending on the presence or absence of alcohol abuse, NASH, or hemochromatosis (not shown). Patients that were clinically classified as cured from HBV infection showed significantly reduced hepatic CAD levels when compared to those with ongoing chronic HBV-induced hepatitis (Figure S2f). When we compared CAD expression with respect to sustained virological response to anti-HCV therapy, we found no significant difference to chronically active cases (Figure S2h). However, small numbers preclude a meaningful interpretation in this particular question. Apart from differences with respect to etiology, we found that women showed lower CAD expression in the surrounding liver tissue (Figure S2I), whereas no significant difference with respect to gender was detected in HCC tissue.

In addition, CAD expression in HCC tissue correlated with proliferation rate (Ki67, R = 0.37, *p* = 6.72 × 10^−19^), alpha fetoprotein (AFP) serum levels (R = 0.32, *p* = 1.5 × 10^−11^), AFP immunoreactivity (R = 0.39, *p* = 3.61 × 10^−20^), active caspase 3 staining (R = 0.17, *p* = 7.35 × 10^−5^), heat-shock protein 70 (HSP70), and glypican 3 (GPC3) immunoreactivity (R = 0.26, *p* = 1.43 × 10^−8^ and R = 0.24, *p* = 1.53 × 10^−9^). Tumors with high CAD expression additionally displayed significantly higher expression of the transcription factor ZEB1 (Figure S2j), which has been implied in cell motility and intrahepatic metastasis [30] and has been described as a marker of so-called epithelial to mesenchymal transition in multiple types of tumors [31]. In line with this, the proportion of ZEB1-positive HCCs was significantly higher among HCCs with high CAD expression (58.9% (43/73)) compared to those with low CAD expression (39.7% (180/453), *p*-value < 0.005).

### 2.3. CPS1 Is Downregulated in Hepatocellular Carcinoma, Further Reduced in Recurrent Tumors and Metastases, and Predicts Prognosis

**Figure 3:**
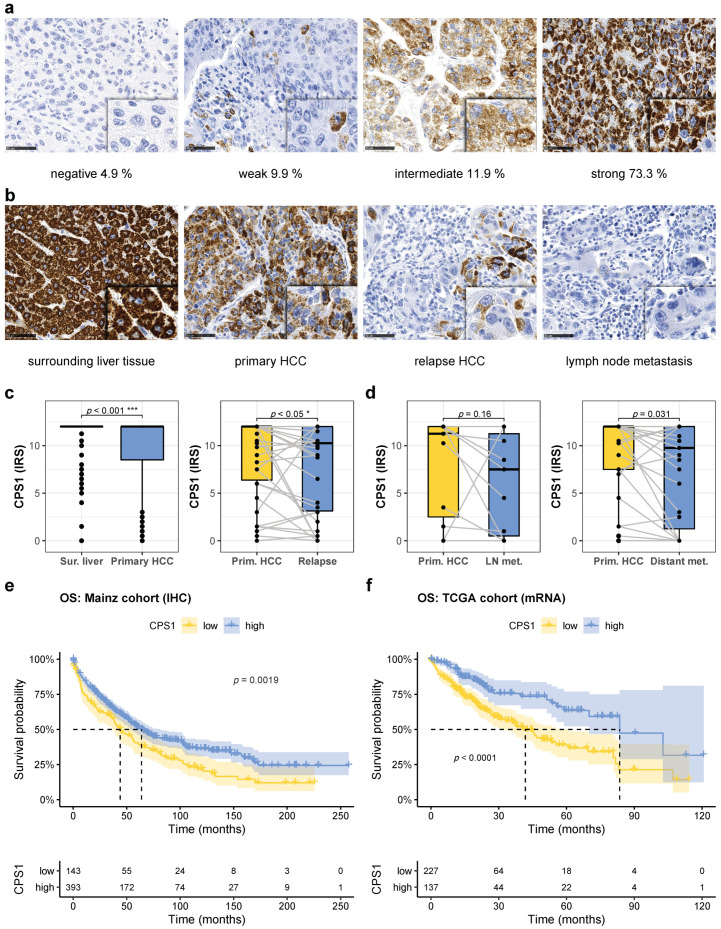
CPS1 is downregulated in primary hepatocellular carcinoma, further reduced during cancer progression, and predicts prognosis. (**a**) Representative images of immunohistochemical staining of HCCs with different expression levels of CPS1. The percentage of tumors with the indicated expression level are shown. Scale bar: 50 µm. (**b**) Decreased expression of CPS1 during tumor progression. Immunostainings for CPS1 of surrounding liver tissue, primary and relapse HCC, and lymph node metastasis of one individual patient. Scale bar: 50 µm. (**c**) Quantitative analysis of CPS1 expression in primary HCC compared to non-neoplastic surrounding liver tissue (left panel, surrounding liver: *n* = 533, HCC: *n* = 536) and in recurrent HCC compared to primary HCC (right panel, *n* = 38). (**d**) Quantification of CPS1 expression in primary HCC compared to lymph node metastasis (left panel, *n* = 11) and distant metastasis (right panel, *n* = 27). (**e**) Kaplan–Meyer plot displaying overall survival with respect to high and low CPS1 expression as detected by immunohistochemistry (HR 1.45 for low CPS1 expression, 95% confidence interval 1.15–1.84, *p* = 0.0019). (**f**) Analysis of overall survival with respect to high and low expression of CPS1 mRNA in the TCGA cohort (HR 2.19, 95% confidence interval 1.48–3.26, *p* ≤ 0.0001). For all analyses * denotes *p* ≤ 0.05, *** p ≤ 0.001.

To further analyze UC dysregulation and pyrimidine synthesis in HCC, the key enzyme catalyzing the initial and rate-limiting step of the UC, CPS1, was investigated. Reduced expression of CPS1 may favor glutamine usage by CAD providing substrates for de novo pyrimidine synthesis and thus favoring proliferation [15]. To test whether CPS1 is of clinical and prognostic relevance, we used the well-established hepatocyte paraffin antigen 1 antibody, which has previously been demonstrated to recognize CPS1 [12], and detected CPS1 by immunoblotting at the predicted molecular weight of 165 kD (Figure S1a). In cultured Huh7 und HepG2 cells, we did not detect CPS1 expression, neither by immunoblot nor immunocytochemistry (Figures S1a,c and S5). CPS1 was not immunohistochemically detectable in 4.9% of the HCCs (IRS = 0), whereas 9.9% displayed weak (IRS = 1–4), 11.9% intermediate (IRS = 5–8), and 73.3% strong (IRS = 9–12) CPS1 immunoreactivity (Figure 3a). CPS1 was downregulated in HCC tissue when compared to the surrounding non-neoplastic liver tissue (Figure 3b,c, left panel), and was further downregulated in recurrent HCCs and distant HCC metastases (Figure 3b–d), while lymph node metastases displayed a trend towards lower CPS1 expression (Figure 3b,d). When we restricted expression analysis to early, most probably true recurrences (<2 years, see above), CPS1 expression was significantly reduced when compared to the primary HCC as well (Figure S2b). Low CPS1 protein expression (IRS ≤ 8.75) in primary HCCs predicted decreased overall survival (Figure 3e). This finding was also confirmed on the mRNA expression level in the TCGA cohort of HCC patients (Figure 3f).

Concerning the regulation of CPS1, it has previously been shown that increased DNA-methylation of the CPS1 promoter in HCC results in decreased CPS1 expression [14,15]. When we analyzed the TCGA dataset, we found that CPS1 mRNA expression is indeed strongly correlated to DNA methylation of the CPS1 locus (R = 0.7, *p* = 1.30 × 10^−54^). CPS1 protein expression levels therefore seem to parallel decreased mRNA levels upon DNA methylation. Furthermore, CPS1 has been demonstrated to be transcriptionally regulated by serine/threonine kinase 11 (encoded by STK11) [32], Y-box binding protein-1 (YBX1), CCAAT enhancer-binding protein-alpha (CEBPA) [33], and hepatocyte nuclear factor 3β (FOXA2) [34]. However, when we analyzed the TCGA dataset, we did not find a significant correlation of CPS1 to STK11 and CEBPA. CPS1 and YBX1 were only weakly inversely correlated (R = −0.13, *p* = 0.01); CPS1 and FOXA2 also showed a weak correlation (R = 0.14, *p* = 8.168 × 10^−3^). While keeping in mind the limitation of these correlational analyses, these known regulators of CPS1 expression are not obviously involved in HCC.

### 2.4. Low CPS1 Expression Is Associated with Unfavorable Prognostic Factors

**Figure 4:**
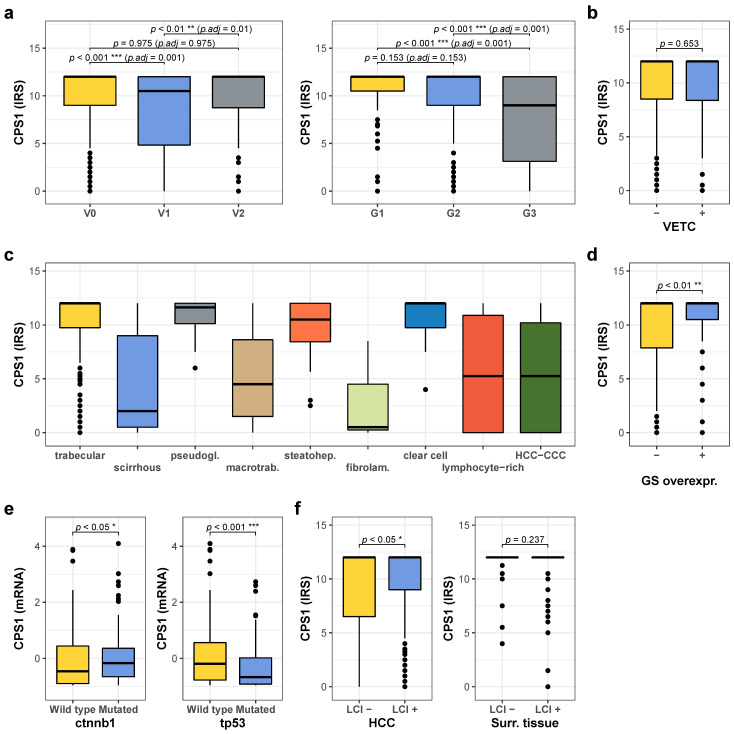
Comparison of CPS1 expression with clinicopathological and molecular parameters. (**a**) Quantification of CPS1 expression detected by immunohistochemistry with respect to vascular invasion (left panel, V0: *n* = 347, V1: *n* = 109, V2: *n* = 80) and tumor grade (right panel, G1: *n* = 100, G2: *n* = 266, G3: *n* = 115). (**b**) Quantification of CPS1 expression in relation to absence or presence of the VETC pattern (VETC-: *n* = 440, VETC+: *n* = 95). (**c**) Quantification of CPS1 expression according to morphological tumor subtype (patient numbers: trabecular: 419, scirrhous: 5, pseudogl.: 18, macrotrab.: 40, steatohep.: 22, fibrolam.: 3, clear cell: 7, lymphocyte-rich: 4, HCC-CCC: 9. (**d**) CPS1 expression levels in relation to absence or presence of GS overexpression as a marker for CTNNB1 mutations/activated WNT signaling (GS neg.: *n* = 423, pos: *n* = 110). (**e**) Quantification of CPS1 mRNA in the TCGA cohort with respect to mutational status to CTNNB1 (left panel, wild type: *n* = 264, mutated: *n* = 96) and TP53 (right panel, wildtype: *n* = 249, mutated 111). (**f**) Quantitative analysis of CPS1 expression in tumor tissue (left panel, no cirrhosis: *n* = 193, cirrhosis: *n* = 343) and in the surrounding liver tissue (right panel, no cirrhosis: *n* = 187, cirrhosis: *n* = 346) with respect to absence of presence of liver cirrhosis). For all analyses * denotes *p* ≤ 0.05, ** *p* ≤ 0.01, *** p ≤ 0.001.

CPS1 expression was then correlated with further clinicopathologic and molecular parameters. Low CPS1 expression in HCC tissue was significantly more often found in female patients and in non-cirrhotic livers, and was associated with higher BCLC stage and with microvascular, but not macrovascular invasion (for clinicopathologic features with respect to low and high CPS1 expression see Table 2). In this line, tumors with microvascular invasion and G3 tumor grade showed lower CPS1 levels (Figure 4a). There was no association of CPS1 expression with the VETC pattern as determined by CD34 staining (Figure 4b). Furthermore, HCC of macrotrabecular-massive, scirrhous, and lymphocyte-rich subtypes, as well as fibrolamellar carcinomas and mixed HCC-CCC tumors had generally lower CPS1 expression (Figure 4c). HCCs with GS overexpression as a marker for CTNNB1 mutations/activation of WNT signaling displayed significantly higher CPS1 protein levels (Figure 4d), and CTNNB1-mutated tumors of the TCGA cohort exhibited high CPS1 mRNA expression (Figure 4e, left panel), whereas mutations of the TP53 gene were associated with lower CPS1 expression (Figure 4e, right panel). In addition, HCCs in a non-cirrhotic liver displayed lower CPS1 expression levels (Figure 4f, left panel). In the surrounding tissue, no difference in CPS1 expression was observed dependent on the presence or absence of cirrhosis (Figure 4f, right panel). We did not detect differences of CPS1 expression with respect to alcohol abuse, NASH, HBV or HCV infection, or hemochromatosis, neither in HCC tissue nor surrounding liver tissue (not shown). In addition, CPS1 expression in HCC tissue inversely correlated with proliferation rate (R = −0.27, *p* = 1.31 × 10^−10^), AFP serum levels (R = −0.23, *p* = 2 × 10^−6^), AFP immunoreactivity (R = −0.25, *p* = 4.5 × 10^−9^), cytokeratin 19 (CK19) immunoreactivity (R = -0.26, *p* = 2.64 × 10^−9^), and CAD expression (R = −0.16, *p* = 0.000316). Additionally, we unraveled a weak but significant correlation to urea blood levels (R = 0.10, *p* = 0.031). Tumors with low CPS1 expression may therefore divert nitrogen in the form of glutamine into tumor pyrimidine synthesis via CAD, instead of catabolizing it into urea by the urea cycle, which has been proposed by Lee et al [6]. Furthermore, tumors with low CPS1 expression displayed significantly higher expression of the transcription factor ZEB1 (Figure S2k), which has been implied in cell motility and intrahepatic metastasis [30,31]. In this line, the proportion of ZEB1-positive HCCs was significantly higher among HCCs with low CPS1 expression (54.6% (77/141)) compared to those with high CPS1 expression (37.6% (145/385), *p*-value < 0.001).

### 2.5. Combined Immunoreactive Score of CAD and CPS1 Improves Prognostic Prediction and Is Particularly Predictive in Early Stage HCC without Vascular Invasion

**Figure 5:**
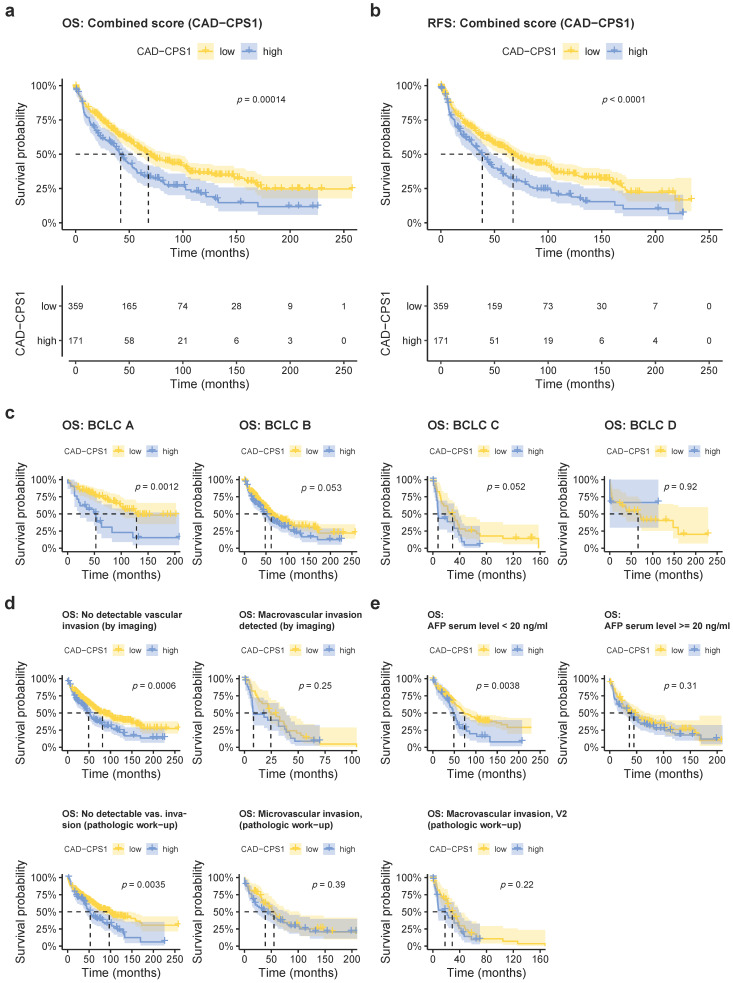
Combining the immunoreactive scores of CAD and CPS1 improves prognostic prediction and is particularly predictive in early stage HCC without vascular invasion. (**a**) Kaplan–Meyer plot showing overall survival rates in patients with respect to a high or low combined CAD-CPS1 score. (**b**) Kaplan–Meyer plot showing recurrence-free survival rates in patients with respect to a high or low combined CAD-CPS1 score. (**c**) Analysis of overall survival in relation to BCLC stage (BCLC A, low: *n* = 76, high = 21; BCLC B, low: *n* = 216, high *n* = 122; BCLC C, low: *n* = 36, high *n* = 25, BCLC D, low: *n* = 31, high: *n* = 3). (**d**) Analysis of overall survival in relation to macrovascular invasion detected by preoperative imaging (upper two panels, no vascular invasion, low: *n* = 322, high: *n* = 144; with vascular invasion, low: *n* = 37, high: *n* = 27) and micro- and macrovascular invasion detected during pathologic work-up (lower three panels, V0, low = 259, high: *n* = 83; V1, low = 53, high = 56; V2, low: *n* = 47, high: *n* = 32). (**e**) Overall survival in relation to AFP serum level (AFP < 20 ng/mL, low: *n* = 198, high: *n* = 49; AFP ≥ 20 ng/mL, low: *n* = 93, high: *n* = 84).

As both dysregulation of CAD and CPS1 have been shown to result in urea cycle dysregulation [6], we hypothesized that combining CAD and CPS1 scores would improve prognostic prediction, as alterations in both enzymes should result in additive biological effects beneficial to tumor growth. In HCC cells, low CPS1 expression may lead to increased shunting of glutamine to CAD, resulting in increased de novo pyrimidine synthesis. Therefore, we subtracted the immunoreactive score for CPS1 from the score for CAD and correlated the combined score with clinicopathological data (Table S2). As expected, a high combined score (>−5.875) was associated with decreased overall and recurrence-free survival rates (Figure 5a,b). When we calculated a combined score for the independent TCGA dataset and analyzed overall survival, we obtained similar results (Figure S4e). A high combined IRS score resulted in more pronounced effects on survival and lower *p* values in the univariate analysis when compared to the single parameters (Figure 5a,b and Table S2), arguing in favor of an additive effect in case of dysregulation of both enzymes. In line with this, the area under the receiver operating characteristic (ROC) curve for 5-year overall survival prediction was increased with the combined score when compared to the single CPS1 or CAD values, although diagnostic prediction solely based on the combined score throughout all BCLC stages was not very accurate (Figure S3a). In the TCGA cohort, combining both scores did not significantly improve accuracy (Figure S4f), which may be related to the fact that protein levels may not exactly parallel mRNA levels. In multivariate analysis, besides established prognostic factors such as macrovascular invasion, BCLC stage, Eastern Cooperative Oncology Group (ECOG) score, and age, the combined CAD–CPS1 score proved to be a statistically independent prognostic factor (Table 3). As the BCLC staging system is the most widely used algorithm for treatment allocation and determination of prognosis of patients with HCC [2], we also performed subgroup analyses stratified according to BCLC stage, and found a significant prognostic effect of the combined score on overall survival only in the BCLC A group, although in BCLC B and C patients, a strong trend was observed (*p* = 0.053 and 0.052, respectively) (Figure 5c). Prediction of 5-year survival was more accurate in the BCLC A group (Figure S3b) when comparing the area under the ROC curve to all BCLC stages (Figure S3a). Furthermore, the combined score only predicted reduced survival in patients without macrovascular invasion detected by imaging (Figure 5d, upper row), and without histologically detectable micro- or macrovascular invasion (Figure 5d, lower row). In addition, the combined score lost its predictive effect in patients with elevated AFP serum levels (Figure 5e) and in tumors with a size of the largest lesion of more than 5 cm (Figure S4c,d). We also found micro- and macrovascular invasion, G3 tumor grade, and VETC pattern to be significantly associated with an increased combined score (Figure S3c–e). Additionally, we detected a significantly higher combined score in primary tumors compared to the surrounding tissue (Figure S3f), and significantly higher levels in recurrent HCCs and distant metastases when compared to the primary tumors (Figure S3g,i). In lymph node metastases, a trend towards higher combined scores was observed (Figure S3h). Finally, we also detected reduced overall and recurrence-free survival rates in the subgroup of patients that underwent liver transplantation (Figure S4a,b).

## 3. Discussion

Several metabolic alterations are frequently found in malignant tumors, such as changes in nutrient uptake and acquisition, and reprogramming of intracellular metabolic pathways. The best known example is the increase in glucose utilization by tumors in comparison to normal tissues, referred to for almost 100 years as the Warburg effect [3]. Another more recently appreciated protumorigenic metabolic program of several cancer types, including HCC, is UC dysregulation, as it has been shown to constitute a survival advantage of tumor cells, and implicates unfavorable prognosis [5,6,16]. Lee et al. have demonstrated that UC dysregulation is a prevalent finding in malignant tumors and results in a shift of nitrogen for pyrimidine synthesis over its catabolism to urea, which in turn leads to increased proliferation rates and an increased pyrimidine-to-purine ratio in several tumors, including HCC [6]. In this study, we investigated the role of two key enzymes involved in de novo pyrimidine synthesis upon UC dysregulation, CAD and CPS1. Here, we provide first evidence that CAD protein is widely expressed among HCCs and is induced during tumor progression. High CAD expression levels are associated with poor prognosis of HCC patients in our cohort as well as in the independent, publicly available TCGA dataset. Additionally, we reproduced data gained in previously published studies on CPS1 [16,35] in a large and clinicopathologically well-characterized patient cohort, including various etiologies and also different tumor stages from early to advanced HCC, showing that CPS1 protein is downregulated in HCC and associated with unfavorable prognosis. This finding also held true when analyzing the independent TCGA cohort of HCC patients. Additionally, we confirmed published data on the use of the HepPar1 antibody [36], demonstrating that a significant proportion of HCCs of the investigated cohort (4.9%) stained negative for CPS1, which represents a potential diagnostic pitfall in histopathology. Furthermore, we demonstrated that CPS1 protein is further downregulated during tumor progression, such as in recurrent tumors and distant metastases. Finally, we extended our analysis to build a combined score of both biomarkers, CAD and CPS1, which improves diagnostic prediction, proves to be an independent prognostic factor in multivariate analysis, and suggests an additive effect of dysregulation of both key enzymes in pyrimidine synthesis on patient survival.

The combined score was particularly predictive in early-stage tumors as demonstrated in subgroup analyses according to BCLC stage and tumor size. The BCLC system, despite being included in clinical guidelines in the US and Europe, has recently been challenged, since non-adherence to treatment recommendations has been reported to result in similar or sometimes even better outcomes [37,38,39,40]. The Hong Kong Liver Cancer (HKLC) classification has been proposed as an alternative to the BCLC system, which may result in a better predictive ability of OS and improved treatment stratification [41,42,43]. In any case, it is increasingly appreciated that combining biomarkers with tumor size and multiplicity may discriminate prognosis better than using tumor burden alone [2,44,45]. Alterations in the combined CAD–CPS1 score may represent such an additional diagnostic tool that can refine prognostic stratification when combined with tumor burden.

From a mechanistic point of view, we found a strong correlation of CPS1 mRNA and methylation of the CPS1 locus, which obviously translates in reduced protein levels in HCC. The specific underlying molecular mechanisms of this regulatory process are currently not known. CAD may be subject to DNA methylation in HCC and may be regulated by MYC and estrogen receptor, but this topic awaits further studies. Importantly, CAD expression is mainly coupled to proliferation [9], which is also in line with the fact that CAD correlated with proliferation rate (Ki67) in our cohort. Furthermore, in our data, as well as in the TCGA dataset, we found CAD to negatively correlate with CPS1. Interestingly, urea cycle dysregulation at different stages has been shown to alter CAD expression [6]. CPS1 downregulation may therefore result in increased CAD expression in HCC cells. Additional complexity is added by the fact that both CAD and CPS1 have also been demonstrated to be regulated post-translationally [46,47,48,49].

Alterations of both enzymes as well as of the combined score in HCC tissue were associated with higher tumor grade, vascular invasion, and increased proliferation rate. The fact that the combined score was only predictive in tumors without detectable vascular invasion indicates that the major underlying mechanism of increased tumor aggressiveness may indeed be increased cancer cell invasion in vessels. In fact, when we analyzed the subgroup of patients without detectable macrovascular invasion (by imaging), 16.9% of patients with a combined score below the determined prognostic cut-off presented with microvascular invasion during pathologic work-up (Table S2), whereas tumors with a score above the cut-off displayed microvascular invasion in 40.3% of cases. When adding the combined score to the multivariate model, the VETC pattern and macrotrabecular-massive tumor subtype, independent predictors of unfavorable outcome also associated with vascular invasion [26,28], lost their significant predictive power, which also argues in the same direction. Interestingly, the combined score is only predictive in AFP-negative tumors, which account for a considerable proportion of all HCCs, especially small tumors [50,51]. AFP positivity is also known to be associated with an increased risk for vascular invasion, which may be the reason why prediction of prognosis by the combined score does not work well in AFP-positive HCCs [52,53]. From a mechanistic point of view, high expression of CAD and low expression of CPS1 were associated with increased activation of the transcription factor ZEB1, which has been demonstrated to regulate cell motility, epithelial to mesenchymal transition, and intrahepatic metastasis [30]. Our observation that CAD levels were significantly higher and CPS1 levels lower in HCCs in non-cirrhotic compared to cirrhotic livers may be explained by the fact that in our cohort, high-grade tumors, which we found to strongly express CAD, were overrepresented among the HCCs that had developed in non-cirrhotic livers. Whether increased CAD expression in HCCs of obese patients represents a specifically regulated process remains to be determined.

We have also made interesting observations concerning the expression of CAD in the non-neoplastic liver tissue. CAD seems to be actively regulated during HBV infection, with increased expression in hepatocytes during chronic HBV infection compared to other etiologies, which is in line with previously published data showing an upregulation of CAD and increased CAD-dependent pyrimidine synthesis upon infection with human cytomegaly virus [54]. Correspondingly, in patients that had eliminated the HBV virus in the past, hepatic CAD expression levels were significantly lower than in ongoing chronic hepatitis B. In contrast to these findings, CAD expression was reduced in chronic HCV infection. These differences in regulation of CAD expression upon HBV and HCV infection suggest molecular differences in the host response to these two particular viruses. Interestingly, CAD has recently been identified as a novel host factor and antiviral target in hepatitis D infection [55].

In terms of feasibility, in our hands, semiquantitative scoring of CAD and CPS1 immunoreactivity was adapted in analogy to the scoring of hormone receptor and HER2neu expression, which is part of routine histopathological diagnostics of breast cancer. In TMA core biopsies, the respective semiquantitative scoring was well reproducible and easy to accomplish. Moreover, staining with the HepPar1 antibody is in many cases performed anyway in differential diagnosis of liver cancer. Taken together, determining the combined CAD–CPS1 score in liver biopsies also seems feasible during routine histopathological analysis and may provide useful additional information on tumor biology, clinical course, and prognosis. It is undoubtedly highly clinically relevant to identify tumors with a high risk of vascular invasion at an early stage [52,56,57]. However, a major limitation of this retrospective study is that the HCC cohort is only composed of surgically resected HCCs, and advanced HCCs undergoing palliative treatment may therefore be underrepresented. Interestingly, CPS1 has been used to detect circulating HCC cells in patients [58], indicating a possible use as a diagnostic biomarker. However, CPS1 has also been demonstrated to be released from non-neoplastic hepatocytes upon liver damage [59] and upon hepatitis C infection [60], and may therefore lack the necessary specificity for a neoplastic origin, for example when measured in blood. CAD, on the other hand, is quite ubiquitously expressed and would thus lack specificity for a hepatic origin [61]. For evaluation of prognosis, our data provide evidence that both markers may probably be best evaluated in the tumor tissue itself.

## 4. Materials and Methods

### 4.1. Patients and Samples

Tissue samples from 561 HCC patients that underwent tumor resection at the University Medical Center Mainz from 1997 to 2018 were provided by the Tissue Biobank of the University Medical Center Mainz after approval by the local ethics committee (Ethik-Kommission der Landesärztekammer Rheinland-Pfalz, 837.146.17 (10980), as well as addendum 2018-13857_1 to DAR and BKS). Clinical data of HCC patients, including survival, were retrieved from a prospectively populated clinical database at our university medical center [62]. Patient records and information were anonymized and de-identified prior to analysis. The mean duration of follow-up was 55.2 months. Details on the patient cohort are given in Table S1.

### 4.2. Cell Culture

Huh7 and HepG2 cells were cultivated in DMEM (Gibco), supplemented with 10% fetal bovine serum (Sigma-Aldrich, Taufkirchen, Germany) and 1% of 10,000 U/mL penicillin and 10 mg/mL streptomycin (Sigma-Aldrich, Taufkirchen, Germany), at 5% CO2 and 37 °C. Cell lines were tested for mycoplasma contamination on a regular basis.

### 4.3. Immunohistochemistry

A tissue microarray (TMA) containing samples from the patient cohort mentioned above was established, comprising at least two cores of primary tumor and surrounding liver tissue, as well as of relapse tumors, lymph node and distant metastases, and tumor thrombi if available. After antigen retrieval, tissue microarray slides were stained with the respective antibodies (see below). Staining was done using an automated staining system (DAKO Autostainer plus, Agilent Technologies, Santa Clara, CA, USA) and the Dako EnVision FLEX staining system (Agilent Technologies, Santa Clara, CA, USA) in accordance with the manufacturer’s instructions. Prior to image analysis, TMA slides were digitalized using the NanoZoomer-Series Digital slide scanner (Hamamatsu Photonics, Hamamatsu, Japan). Immunoreactivity was either scored semiquantitatively according to Remmele et al. [18], or in the case of Ki67 and CD34, digital image analysis was performed using the HALO platform from Indica Labs (Corrales, NM, USA), including the TMA module and the CytoNuclear v1.6 module. Missing or erroneous cores (e.g., those with extensive tumor necrosis) were excluded from the analysis. In the case of Ki67, positive nuclei were counted; in the case of CD34, the positively stained area was quantified. The antibodies, dilutions, and antigen retrieval methods used are summarized in Appendix A.

### 4.4. Immunofluorescence

Huh7 and HepG2 cells were seeded on 13 mm coverslips in 12-well plates. After washing with PBS, cells were fixed for 10 min with 3.7% formaldehyde in PBS, subsequently washed with PBS, permeabilized and blocked with 10% FBS, 0.1% Triton X-100 in PBS at 37 °C. After incubation with the primary antibody (anti-CAD, Abcam, Cambridge, UK, ab40765, rabbit monoclonal, 1:100) in blocking solution at RT for 1 h and a brief wash with 0.1% Triton X-100 in PBS, cells were incubated with Alexa Fluor® 488 goat anti-rabbit IgG, MitoView 633 (1:1000), and the nuclear dye DAPI in 1% FBS, 0.1% Triton X-100 in PBS at RT for 1 h. After an additional washing step with 0.1% Triton X-100 in PBS, cells were mounted with MOWIOL, and images were obtained with a Leica SP8 confocal microscope using either a 40× 1.30 NA Oil CS2 HC Plan Apo or a 63× 1.40 NA Oil CS2 HC Plan Apo objective operating at 25 °C.

### 4.5. Immunoblotting

Human HCC and liver tissue and cell culture lysates were homogenized in cell lysis buffer (50 mM Tris, 100 mM NaCl, 15 mM EGTA, 1% Triton X-100, pH = 8), incubated with hot 2× Laemmli buffer at 95 °C for 5 min, and then loaded on SDS-PAGE gels. Proteins were transferred to nitrocellulose membranes, which were blocked with 5% dry-milk, incubated with either anti-CAD (1:1000 dilution) or the HepPar1-antibody (1:100 dilution) overnight at 4° C, and subsequently with HRP-conjugated secondary antibodies for 1–2 h at room temperature. For detection, enhanced chemiluminescence and a digital detection system (Fusion Solo S, Vilber, Eberhardzell, Germany) were used.

### 4.6. Analysis of Publicly Available Data Sets

CAD and CPS1 expression data from the hepatocellular carcinoma cohort compiled by The Cancer Genome Atlas (TCGA) program were downloaded from http://cbioportal.org (accessed on 11 February 2021) and dichotomized by mutational status of the TP53 or CTNNB1 gene [63]. Kaplan–Meier curves dichotomized by high and low CAD or CPS1 expression levels were downloaded from http://proteinatlas.org (accessed on 20 May 2020) [64]. Expression data were log2-transformed before building the combined CAD–CPS1 score.

### 4.7. Statistical Analysis

All statistical analyses were performed within the R environment for statistical computing (version 4.0.3, R Foundation for Statistical Computing, Vienna, Austria) [65]. The non-parametric Mann–Whitney U test was applied to compare differences between two independent groups when dependent variables were either ordinal or continuous. The non-parametric Wilcoxon signed-rank test was used to determine whether two dependent samples were selected from populations having the same distribution. The Kruskal–Wallis test was applied to compare two independent groups, which consist of one dependent scale variable and one explanatory nominal variable with 3 or more levels. Benjamini–Hochberg corrections were applied to reduce the effects of multiple testing and control for the false discovery rate. Categorical variables were compared using the χ2 test or Fisher’s exact test. *p*-values ≤ 0.05 were considered statistically significant. CPS1 and CAD protein expression and the combined CAD–CPS1 score were dichotomized utilizing the Charité Cutoff Finder functions to provide a significant distinction between the high and low expression levels based on survival outcome [66]. Overall survival was calculated as the interval between initial diagnosis and death, regardless of etiology or the last follow-up. Recurrence-free survival was defined as the interval from initial therapy to detection of progression (regardless of the location) or death. Last follow-up or death of other causes were considered censored events. Overall survival and recurrence-free survival were calculated by the Kaplan–Meyer method, and differences were evaluated by the log-rank test. Uni- and multivariate Cox regression analysis was conducted for 561 patients with a mean survival time of 55.2 months using the functions coxph from the R package survival (version 3.2.7, R Foundation for Statistical Computing, Vienna, Austria). In order to create a significant multivariate Cox model, a stepwise variable selection was performed by employing the stepwise backward model selection by the Akaike information criterion (AIC) method from the R package MASS (version 7.3.53, R Foundation for Statistical Computing, Vienna, Austria) [67]. At last, the combined CAD–CPS1 score was added to the statistical model. ROC curves were used to determine the biomarker potential of CPS1, CAD, and CAD–CPS1 relative to 5-year survival in HCC.

## 5. Conclusions

HCC represents a malignancy with high prevalence and high mortality worldwide. Besides serum levels of α-fetoprotein, prognostic tissue biomarkers have not been implemented in clinical decision making so far [2]. There is an urgent need for new treatment targets and biomarkers to provide additional information for patient risk stratification and molecular-guided targeted therapy. A major hindrance in this respect is that according to current guidelines, liver biopsy for the diagnosis of non-resectable, advanced HCC in a context of liver cirrhosis is facultative, so data on biomarker expression in HCCs treated with palliative intent are missing. Correlation with clinical and histologic findings indicates functional relevance and highlights the combined CAD–CPS1 score as an independent prognostic biomarker, especially in early tumor stages and in AFP-negative tumors. Interestingly, it has recently been demonstrated that targeting pyrimidine synthesis at different levels, including inhibiting CAD, reduced the growth of glioblastoma cells in a rodent model [7]. Further studies are needed to ascertain whether CAD, CPS1, and the combined CAD–CPS1 score are of predictive value, and whether new therapeutic agents inhibiting pyrimidine synthesis may also be a treatment option in HCC patients.

## Figures and Tables

**Table 1:** Clinicopathologic data associated with high and low immunoreactive scores of CAD.

Characteristic		CAD Low	CAD High	*p*-Value ^2^
		*N* = 459 (86%) ^1^	*N* = 74 (14%) ^1^	
Median age in years (range)		64.7 (56.2, 70.7)	62.6 (56.8, 72.3)	0.9
Median tumor size in mm		40.0 (25.0, 78.0)	45.0 (27.5, 96.5)	0.2
Number of tumors				0.6
	1	244 (63%)	44 (67%)	
	≥2	146 (37%)	22 (33%)	
Gender				0.042
	Male	368 (80%)	51 (69%)	
	Female	91 (20%)	23 (31%)	
Etiology of liver disease				
	Alcohol abuse	144 (31%)	14 (19%)	0.041
	HCV	93 (20%)	14 (19%)	>0.9
	HBV	88 (19%)	11 (15%)	0.5
	NASH	34 (7.4%)	8 (11%)	0.4
	Hemochromatosis	20 (4.4%)	4 (5.4%)	0.8
	Unknown/Other	111 (24%)	15 (20%)	0.6
BCLC				0.006
	A	88 (19%)	10 (14%)	
	B	290 (63%)	50 (68%)	
	C	47 (10%)	14 (19%)	
	D	34 (7.4%)	0 (0%)	
ECOG PST				0.7
	0–1	438 (96%)	72 (100%)	
	2	9 (2.0%)	0 (0%)	
	3	5 (1.1%)	0 (0%)	
	4	6 (1.3%)	0 (0%)	
Liver cirrhosis				0.3
	Absent	161 (35%)	31 (42%)	
	Present	298 (65%)	43 (58%)	
Child–Pugh score				0.024
	A	164 (52%)	30 (61%)	
	B	119 (38%)	19 (39%)	
	C	34 (11%)	0 (0%)	
Portal vein thrombosis		48 (10%)	16 (22%)	0.011
Vascular invasion				<0.001
	Absent	314 (68%)	30 (41%)	
	Micro	83 (18%)	26 (35%)	
	Macro	62 (14%)	18 (24%)	
Tumor grading				<0.001
	G1	95 (23%)	4 (6.0%)	
	G2	229 (56%)	36 (54%)	
	G3	88 (21%)	27 (40%)	
Macrotrabecular subtype				0.082
	no	420 (93%)	64 (86%)	
	yes	31 (6.9%)	10 (14%)	
VETC				0.2
	negative	382 (83%)	56 (76%)	
	positive	77 (17%)	18 (24%)	
GS overexpression				0.3
	no	360 (79%)	63 (85%)	
	yes	98 (21%)	11 (15%)	

^1^ Statistics presented: median (IQR); *n* (%). ^2^ Statistical tests performed: Wilcoxon rank-sum test; chi-square test of independence; Fisher’s exact test.

**Table 2:** Clinicopathologic data associated with high and low immunoreactive scores of CPS1.

Characteristic		CPS1 Low	CPS1 High	*p*-Value ^2^
		*N* = 143 (27%) ^1^	*N* = 393 (73%) ^1^	
Median age in years (range)		64.0 (56.2, 69.3)	64.8 (56.5, 71.3)	0.2
Median tumor size in mm		48.0 (29.0, 90.8)	38.0 (24.0, 78.0)	0.039
Number of tumors				>0.9
	1	76 (63%)	214 (64%)	
	≥2	44 (37%)	123 (36%)	
Gender				0.054
	Male	104 (73%)	318 (81%)	
	Female	39 (27%)	75 (19%)	
Etiology of liver disease				
	Alcohol abuse	40 (28%)	121 (31%)	0.6
	HCV	24 (17%)	83 (21%)	0.3
	HBV	30 (21%)	70 (18%)	0.5
	NASH	10 (7.0%)	31 (7.9%)	0.9
	Hemochromatosis	8 (5.6%)	17 (4.3%)	0.7
	Unknown/Other	40 (28%)	88 (22%)	0.2
BCLC				0.014
	A	19 (13%)	79 (20%)	
	B	104 (73%)	239 (61%)	
	C	17 (12%)	44 (11%)	
	D	3 (2.1%)	31 (7.9%)	
ECOG PST				0.7
	0–1	137 (97%)	376 (96%)	
	2	2 (1.4%)	7 (1.8%)	
	3	0 (0%)	5 (1.3%)	
	4	2 (1.4%)	4 (1.0%)	
Liver cirrhosis				0.008
	Absent	65 (45%)	128 (33%)	
	Present	78 (55%)	265 (67%)	
Child–Pugh score				0.11
	A	47 (55%)	146 (52%)	
	B	35 (41%)	105 (37%)	
	C	3 (3.5%)	31 (11%)	
Portal vein thrombosis		16 (11%)	48 (12%)	0.9
Vascular invasion				<0.001
	Absent	76 (53%)	271 (69%)	
	Micro	47 (33%)	62 (16%)	
	Macro	20 (14%)	60 (15%)	
Tumor grading				<0.001
	G1	17 (13%)	83 (24%)	
	G2	64 (49%)	202 (58%)	
	G3	50 (38%)	65 (19%)	
Macrotrabecular subtype				<0.001
	no	113 (79%)	374 (97%)	
	yes	30 (21%)	10 (2.6%)	
VETC				>0.9
	negative	117 (82%)	323 (82%)	
	positive	26 (18%)	69 (18%)	
GS overexpression				0.004
	no	125 (88%)	298 (76%)	
	yes	17 (12%)	93 (24%)	

^1^ Statistics presented: median (IQR); *n* (%). ^2^ Statistical tests performed: Wilcoxon rank-sum test; chi-square test of independence; Fisher’s exact test.

**Table 3:** Univariate and multivariate Cox regression analyses of prognostic factors (overall survival).

Characteristic	Univariate				Multivariate			
	HR	Conf.Low	Conf.High	*p*.Value	HR	Conf.Low	Conf.High	*p*.Value
**Clinical Features**								
Age (≥60 vs. <60)	1.43	1.13	1.80	<0.01	1.44	1.13	1.84	0.003
Sex (male vs. female)	1.07	0.82	1.39	0.64				
Alcohol abuse (tr. vs. f.)	0.87	0.69	1.10	0.25				
NASH (true vs. false)	1.08	0.70	1.65	0.74				
Haemochrom. (tr. vs. f.)	0.70	0.41	1.19	0.19				
HBV (pos. vs. neg.)	0.89	0.67	1.17	0.40				
HCV (pos. vs. neg.)	1.07	0.82	1.40	0.61				
Child–Pugh (B/C vs. A)	0.97	0.74	1.26	0.81				
ECOG (PST1-4 vs. PST0)	1.43	1.13	1.79	<0.01	1.47	1.15	1.87	0.002
BCLC new (B-D vs. A)	1.83	1.35	2.50	<0.001	1.43	1.03	1.98	0.035
AFP (≥ vs. < 20 ng/mL)	1.58	1.13	2.21	<0.01				
Albumin (≥ vs. < 25.75 g/l)	0.56	0.42	0.74	<0.0001				
TNM M (M1 vs. M0)	2.65	1.41	4.99	<0.01				
**Pathologic features**								
Liver cirrhosis (yes vs. no)	0.85	0.68	1.07	0.18				
Grading (G3 vs. G1/G2)	1.50	1.15	1.95	<0.01				
TNM N (N1 vs. N0)	1.68	0.79	3.57	0.17				
VI (micro vs. none)	1.47	1.12	1.93	<0.01	1.17	0.87	1.57	0.309
VI (macro vs. none)	3.18	2.39	4.25	<0.0001	2.57	1.89	3.51	<0.0001
Macrotrabecular Subtype	1.96	1.34	2.85	<0.001	1.43	0.94	2.20	0.098
VETC (pos. vs. neg.)	1.50	1.14	1.99	<0.01	1.32	0.99	1.77	0.055
GS (≥9 vs. <9)	0.77	0.58	1.02	0.07				
CAD–CPS1 (high vs. low)	1.56	1.24	1.96	<0.001	1.37	1.06	1.75	0.014

## Data Availability

No new data were created or analyzed in this study. Data sharing is not applicable to this article.

## Supplementary Materials

The following are available online at https://www.mdpi.com/2072-6694/13/4/744/s1, Figure S1: Expression and localization of CAD and CPS1 in HCC cell lines, HCCs and surrounding liver tissue. (**a**) Detection of CAD and CPS1 expression by immunoblot in extracts isolated from Huh7 and HepG2 cells and from HCC samples from 3 individual patients (HCC 1 to 3) and corresponding surrounding liver tissue of patient 1 (LT1). Actin is shown as a loading control. Huh7 and HepG2 cells show no detectable CPS1 expression. (**b**) Immunofluorescent staining of Huh7 cells for CAD (green). MitoView (purple) labels Mitochondria, DAPI (blue) cell nuclei. Scale bar indicates 25 µm. (**c**) Immuno-histochemical stainings for CAD, CPS1, ZEB1, and Ki67 of Huh7 and HepG2 cells, HCC samples from three individual patients, and surrounding liver tissue of patient 1 (samples correspond to those subjected to immunoblot, see Figure S1a). Figure S2: Comparison of CAD and CPS1 expression with additional clinicopathological parameters. (**a**) Quantification of CAD expression in primary HCCs and in the respective early relapses (<2 years) (*n* = 26, paired analysis). (**b**) Quantification of CPS1 expression in primary HCCs and in the respective early relapses (<2 years) (*n* = 25, paired analysis). (**c**) Quantifi-cation of CAD expression in primary HCCs with respect to absence or presence of obesity (absent: *n* = 450, present: *n* = 76). (**d**) Quantification of CAD expression in primary HCCs with respect to alcohol abuse (false: *n* = 373, true: *n* = 157). (**e**) Quantification of CAD expression in surrounding liver tissue with respect to presence or absence of chronic hepatitis B (false: *n* = 467, true: *n* = 63). (**f**) Quantification of CAD expression in surrounding liver tissue of patients suffering from chronic hepatitis B compared to patients with a history of cured hepatitis B infection (chronic HBV: *n* = 63, cured HBV: *n* = 41). (**g**) Quantification of CAD expression in surrounding liver tissue with respect to presence or absence of chronic hepatitis C (false: *n* = 428, true: *n* = 102). (**h**) Quantification of CAD expression in surrounding liver tissue of patients suffering from chronic hepatitis C compared to patients with a history of cured hepatitis C infection (chronic HBV: *n* = 102, cured HBV: *n* = 5). (**i**) Quantification of CAD expression in surrounding liver tissue according to gender (female: *n* = 117, male: *n* = 413). (**j**) Quantification of ZEB1 expression in primary HCCs according to low vs. high CAD expression (low: *n* = 453, high: *n* = 73). (**k**) Quantification of ZEB1 expression in primary HCCs according to low vs. high CPS1 expression (low: *n* = 141, high: *n* = 385). For all analyses * denotes *p* ≤ 0.05, ** *p* ≤ 0.01, *** *p* ≤ 0.001. Figure S3: Comparison of the combined CAD-CPS1 score with additional parameters. (**a**) ROC curves for 5-year overall survival for CAD expression (left panel, diseased: *n* = 250, alive: *n* = 283), CPS1 expression (middle panel, diseased: *n* = 250, alive: *n* = 286) and the combined score of CAD–CPS1 (right panel, diseased: *n* = 249, alive: *n* = 281) (IHC) in the Mainz cohort. All BCLC stages are considered. (**b**) ROC curves for 5-year overall survival for CAD expression (left panel, diseased: n = 31, alive: *n* = 67), CPS1 expression (middle panel, diseased: *n* = 30, alive: *n* = 68), and the combined score of CAD–CPS1 (right panel, diseased: *n* = 30, alive: *n* = 67) in the BCLC stage A subgroup. (**c**–**e**) Combined CAD–CPS1 score in relation to vascular invasion (V0: *n* = 342, V1: *n* = 109, V2: *n* = 79), tumor grade (G1: *n* = 99, G2: *n* = 263, G3: *n* = 114), and VETC pattern (VETC-: *n* = 435, VETC+: *n* = 95). (**f**) Combined CAD–CPS1 score in surrounding liver tissue and primary HCC (both *n* = 502). G/H/I: Combined CAD–CPS1 score in primary HCC compared to relapse HCC (**g**, *n* = 38), lymph node metastases (**h**, *n* = 11), and distant metastases (**i**, *n* = 27). For all analyses * denotes *p* ≤ 0.05, ** *p* ≤ 0.01, *** *p* ≤ 0.001. Figure S4: Additional survival analyses with respect to the combined CAD-CPS1 score. (**a**) Overall survival after liver transplan-tation in relation to high or low combined CAD–CPS1 score. (**b**) Recurrence-free survival after liver transplantation in relation to high or low combined CAD–CPS1 score. C/D: Analysis of overall survival in the subgroup of HCCs with a size of the largest lesion <5 cm (**c**) and ≥5 cm (**d**). (**e**) Kaplan–Meyer plot showing overall survival rates in patients of the TCGA cohort with respect to a high or low combined CAD–CPS1 score (mRNA). (**f**) ROC curves for 5-year overall survival for CAD expression (left panel, diseased: *n* = 121, alive: *n* = 244), CPS1 expression (middle panel, diseased: *n* = 120, alive: *n* = 243), and the combined score of CAD–CPS1 (right panel, diseased: *n* = 120, alive: *n* = 243) (mRNA) in the TCGA cohort. Figure S5: Original images of the western blots shown in Figure S1. Densitometric readings are displayed below the band of interest. Table S1: Clinicopathologic data of the patient cohort. Table S2: Clinicopathologic data associated with high and low combined CAD-CPS1 score.

## Appendix A

Antibodies, dilutions and retrieval solutions used for immunohistochemistry

- Anti-AFP (Dako, Hamburg, Germany, #IR500, rabbit polyclonal, ready to use, Tris/EDTA buffer, pH 9 (Dako #8024))
- Anti-CPS1 (HepPar1, Dako, #IR624, mouse monoclonal, ready to use, Tris/EDTA buffer, pH 9)
- Anti-CAD (Santa Cruz, Heidelberg, Germany, #sc-376072, mouse monoclonal, 1:75, Tris/EDTA buffer, pH 9)
- Anti-cleaved Caspase 3 (Cell Signaling, Frankfurt, Germany, #9664, rabbit monoclonal, 1:200, citrate buffer, pH = 6.1 (Dako #GV805))
- Anti-CD34 (Dako, #IR632, mouse monoclonal, ready to use, Tris/EDTA buffer, pH 9)
- Anti-CK19 (Dako #IR615, mouse monoclonal, ready to use, Tris/EDTA buffer, pH 9)
- Anti-Glutaminsynthetase (Roche, Mannheim, Germany, #760-4898, mouse monoclonal, ready to use, cell conditioning solution 1 (Roche, #950-124))
- Anti-Glypican 3 (Roche, #790-4564, mouse monoclonal, ready to use, cell conditioning solution 1 (Roche, #950-124))
- Anti-γH2AX (Cell Signaling, #9718, rabbit monoclonal, 1:200, citrate buffer, pH = 6.1)
- Anti-HSP70 (Santa Cruz, #sc-24, 1:250, cell conditioning solution 1 (Roche, #950-124))
- Anti-Ki67 (Dako, #IR626, mouse monoclonal, ready to use, citrate buffer, pH = 6.1)
- Anti-ZEB1 (Novusbio, Centennial CO, USA, NBP1-05987, rabbit polyclonal, 1:500, Tris/EDTA buffer, pH 9)
