# Supplementary material for: Key Enzymes in Pyrimidine Synthesis, CAD and CPS1, Predict Prognosis in Hepatocellular Carcinoma
Source: Cancers (Basel). 2021 Feb 11;13(4):744. doi: 10.3390/cancers13040744 (PMC7916936; doi:10.3390/cancers13040744)
Supplement: Supplementary file 1 [file cancers-13-00744-s001.pdf]

Supplementary Material: Key Enzymes in Pyrimidine Synthesis, CAD and CPS1, Predict Prognosis in Hepatocellular Carcinoma

Dirk Andreas Ridder, Mario Schindeldecker, Arndt Weinmann, Kristina Berndt, Lana Urbansky, Hagen Roland Witzel, Stefan Heinrich, Wilfried Roth and Beate Katharina Straub

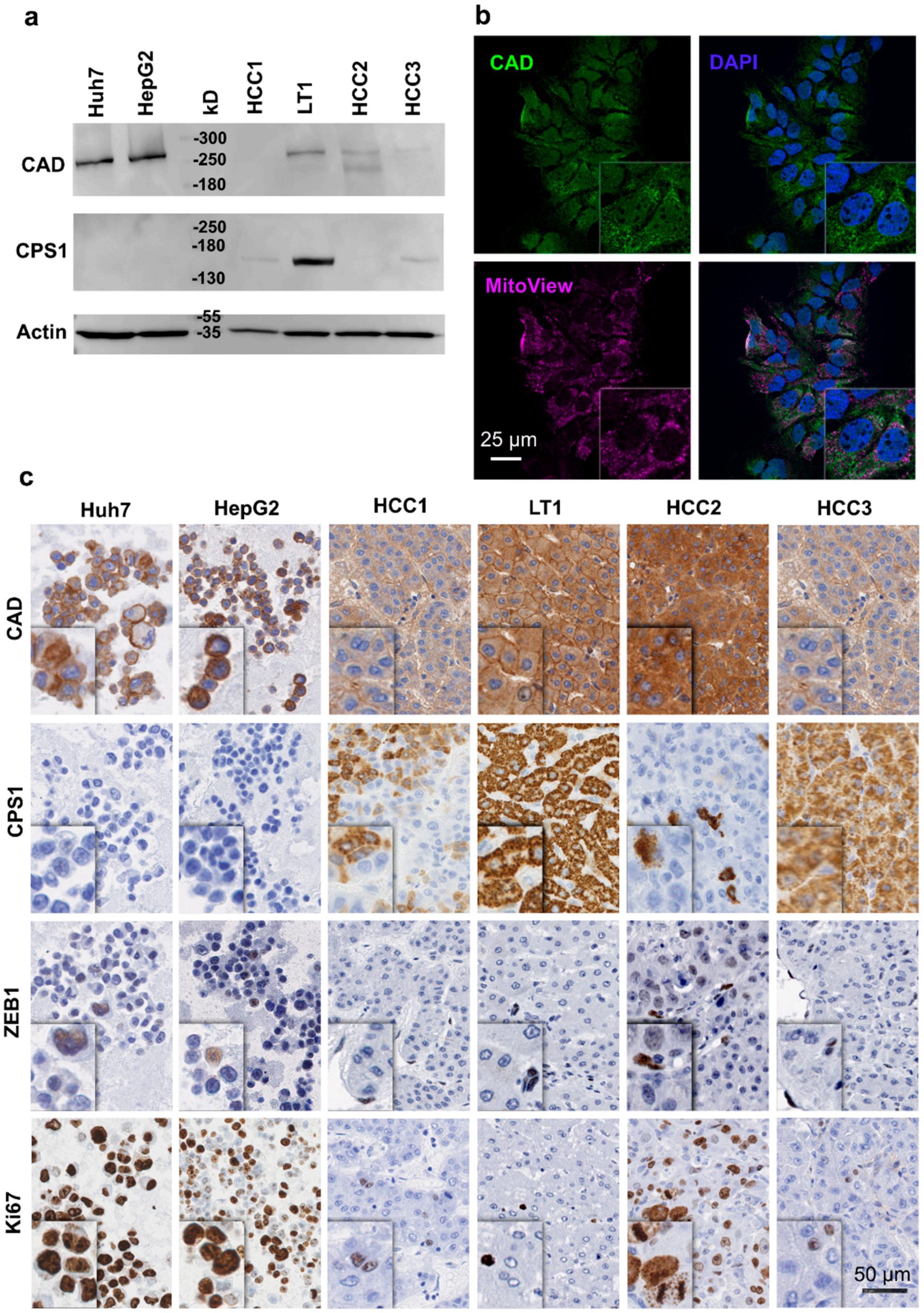

Figure S1. Expression and localization of CAD and CPS1 in HCC cell lines, HCCs and surrounding liver tissue. (**a**) Detection of CAD and CPS1 expression by immunoblot in extracts isolated from Huh7 and HepG2 cells and from HCC

samples from 3 individual patients (HCC 1 to 3) and corresponding surrounding liver tissue of patient 1 (LT1). Actin is shown as a loading control. Huh7 and HepG2 cells show no detectable CPS1 expression. (**b**) Immunofluorescent staining of Huh7 cells for CAD (green). MitoView (purple) labels Mitochondria, DAPI (blue) cell nuclei. Scale bar indicates 25 µm. (**c**) Immunohistochemical stainings for CAD, CPS1, ZEB1, and Ki67 of Huh7 and HepG2 cells, HCC samples from three individual patients, and surrounding liver tissue of patient 1 (samples correspond to those subjected to immunoblot, see Figure S1a).

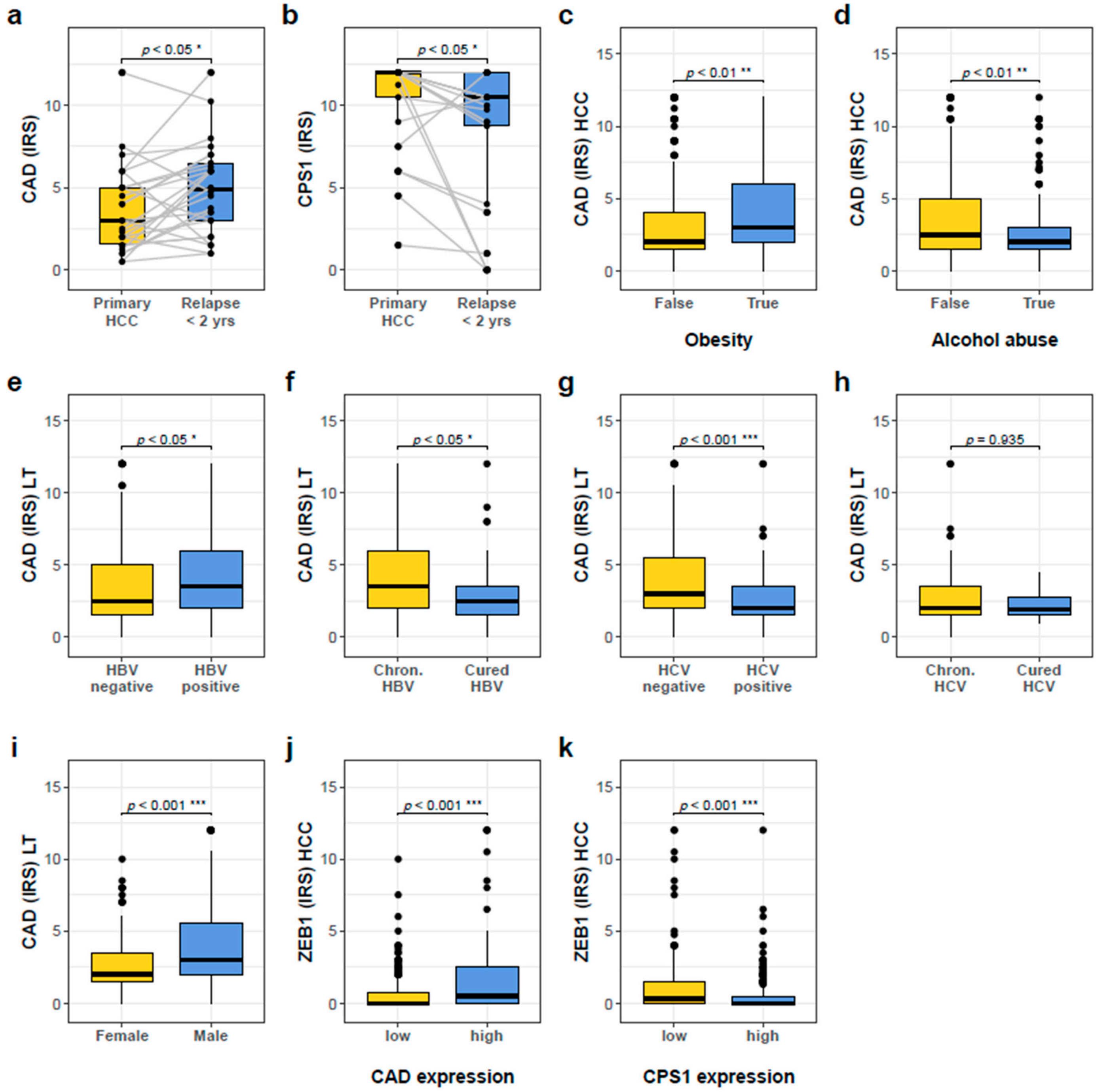

Figure S2. Comparison of CAD and CPS1 expression with additional clinicopathological parameters. (**a**) Quantification of CAD expression in primary HCCs and in the respective early relapses (<2 years) (n = 26, paired analysis). (**b**) Quantification of CPS1 expression in primary HCCs and in the respective early relapses (<2 years) (n = 25, paired analysis). (**c**) Quantification of CAD expression in primary HCCs with respect to absence or presence of obesity (absent: n = 450, present: n = 76). (**d**) Quantification of CAD expression in primary HCCs with respect to alcohol abuse (false: n = 373, true: n = 157). (**e**) Quantification of CAD expression in surrounding liver tissue with respect to presence or absence of chronic

hepatitis B (false: n = 467, true: n = 63). (**f**) Quantification of CAD expression in surrounding liver tissue of patients suffering from chronic hepatitis B compared to patients with a history of cured hepatitis B infection (chronic HBV: n = 63, cured HBV: n = 41). (**g**) Quantification of CAD expression in surrounding liver tissue with respect to presence or absence of chronic hepatitis C (false: n = 428, true: n = 102). (**h**) Quantification of CAD expression in surrounding liver tissue of patients suffering from chronic hepatitis C compared to patients with a history of cured hepatitis C infection (chronic HBV: n = 102, cured HBV: n = 5). (**i**) Quantification of CAD expression in surrounding liver tissue according to gender (female: n = 117, male: n = 413). (**j**) Quantification of ZEB1 expression in primary HCCs according to low vs. high CAD expression (low: n = 453, high: n = 73). (**k**) Quantification of ZEB1 expression in primary HCCs according to low vs. high CPS1 expression (low: n = 141, high: n = 385). For all analyses * denotes $p \leq 0.05$, ** $p \leq 0.01$, *** $p \leq 0.001$.

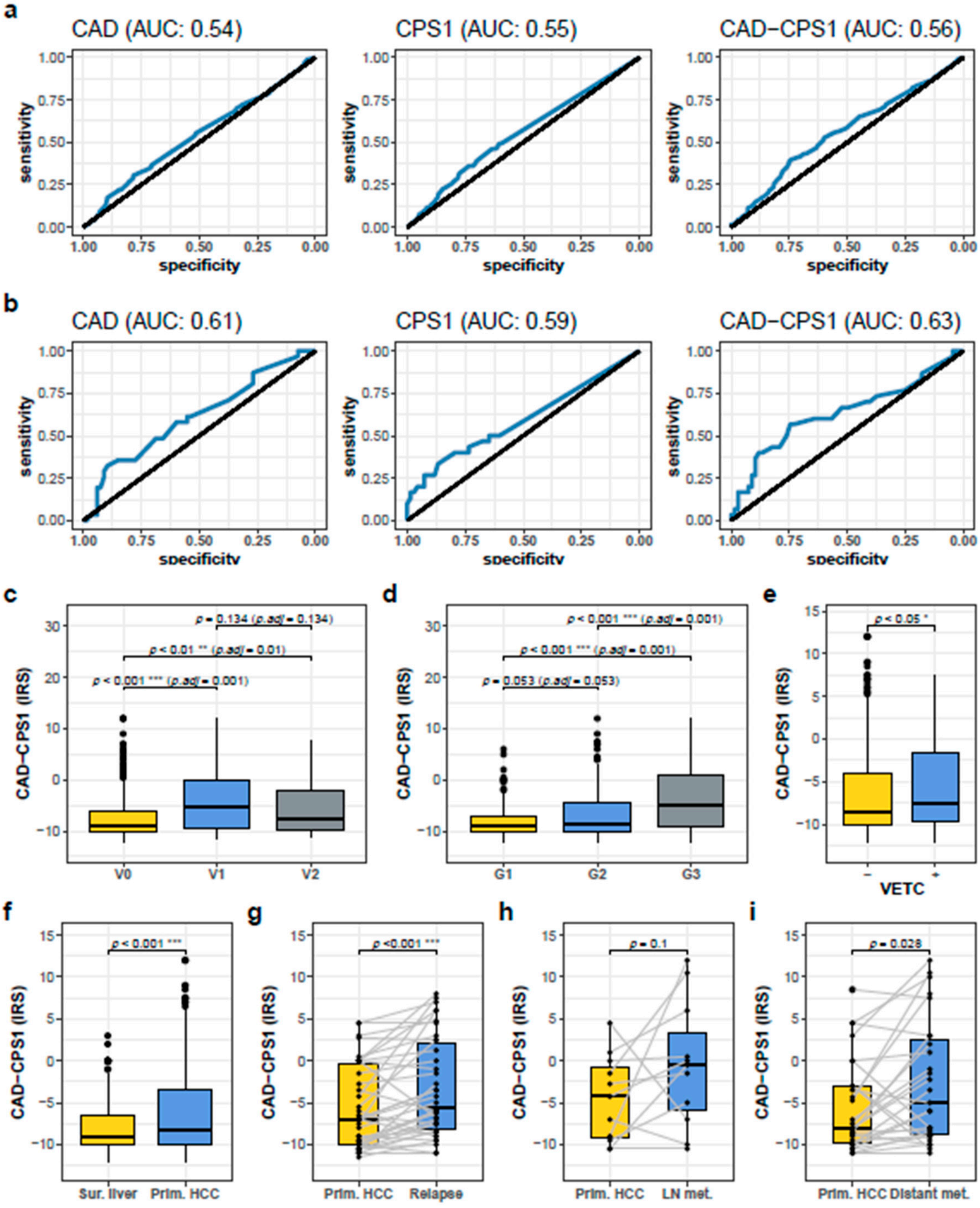

Figure S3. Comparison of the combined CAD-CPS1 score with additional parameters (**a**) ROC curves for 5-year overall survival for CAD expression (left panel, diseased: n = 250, alive: n = 283), CPS1 expression (middle panel, diseased: n = 250, alive: n = 286) and the combined score of CAD–CPS1 (right panel, diseased: n = 249, alive: n = 281) (IHC) in the Mainz cohort. All BCLC stages are considered. (**b**) ROC curves for 5-year overall survival for CAD expression (left panel,

diseased: $n = 31$, alive: $n = 67$), CPS1 expression (middle panel, diseased: $n = 30$, alive: $n = 68$), and the combined score of CAD–CPS1 (right panel, diseased: $n = 30$, alive: $n = 67$) in the BCLC stage A subgroup. (**c**–**e**) Combined CAD–CPS1 score in relation to vascular invasion (V0: $n = 342$, V1: $n = 109$, V2: $n = 79$), tumor grade (G1: $n = 99$, G2: $n = 263$, G3: $n = 114$), and VETC pattern (VETC-: $n = 435$, VETC+: $n = 95$). (**f**) Combined CAD–CPS1 score in surrounding liver tissue and primary HCC (both $n = 502$). G/H/I: Combined CAD–CPS1 score in primary HCC compared to relapse HCC (**g**, $n = 38$), lymph node metastases (**h**, $n = 11$), and distant metastases (**i**, $n = 27$). For all analyses * denotes $p \leq 0.05$, ** $p \leq 0.01$, *** $p \leq 0.001$.

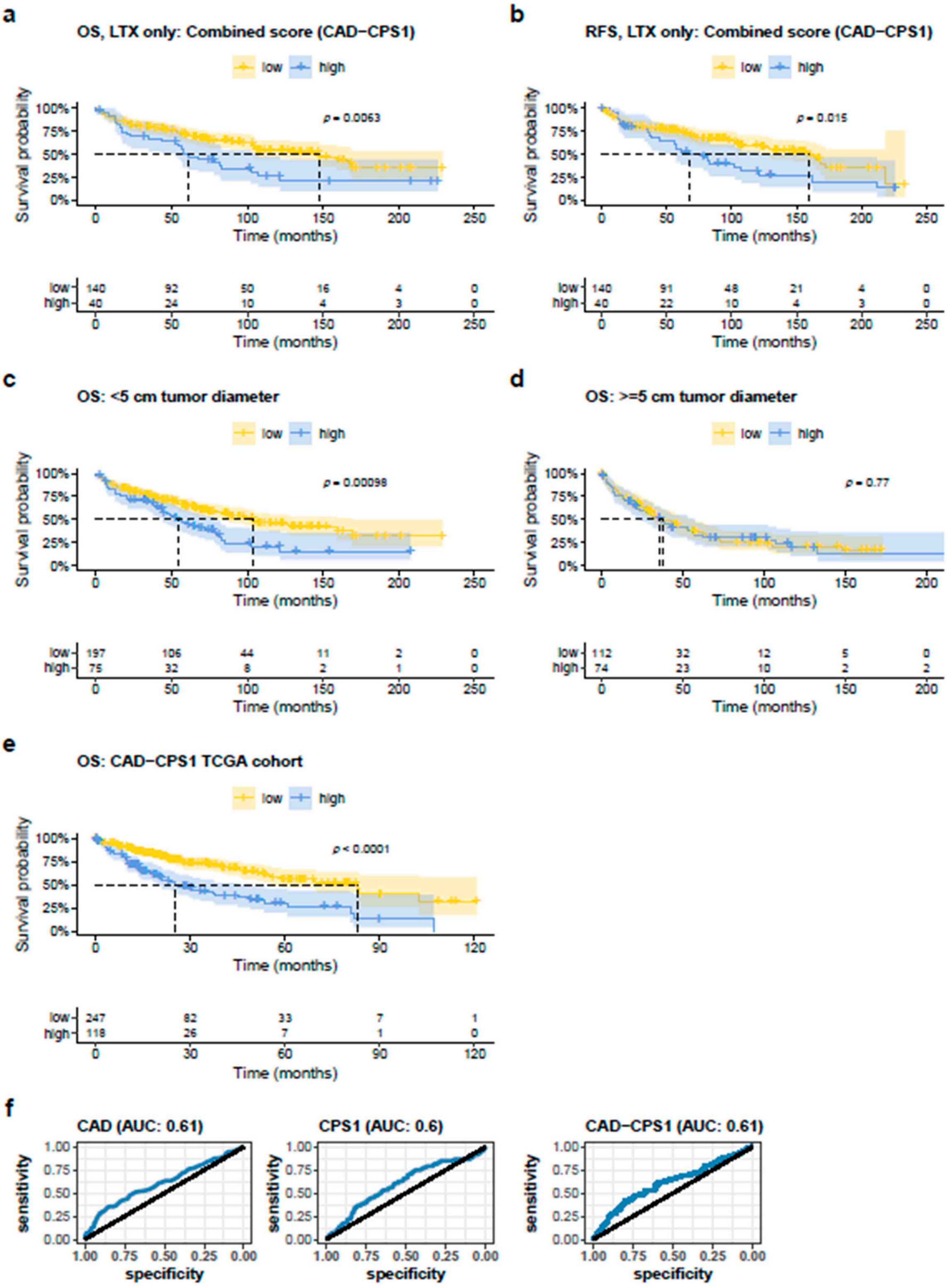

Figure S4. Additional survival analyses with respect to the combined CAD-CPS1 score. (**a**) Overall survival after liver transplantation in relation to high or low combined CAD–CPS1 score. (**b**) Recurrence-free survival after liver trans-

plantation in relation to high or low combined CAD–CPS1 score. C/D: Analysis of overall survival in the subgroup of HCCs with a size of the largest lesion <5 cm (**c**) and ≥5 cm (**d**). (**e**) Kaplan–Meyer plot showing overall survival rates in patients of the TCGA cohort with respect to a high or low combined CAD–CPS1 score (mRNA). (**f**) ROC curves for 5-year overall survival for CAD expression (left panel, diseased: n = 121, alive: *n* = 244), CPS1 expression (middle panel, diseased: *n* = 120, alive: *n* = 243), and the combined score of CAD–CPS1 (right panel, diseased: *n* = 120, alive: *n* = 243) (mRNA) in the TCGA cohort.

Table S1. Clinicopathologic data of the patient cohort.

Characteristic	*N* = 561 [1]
Median age in years (range)	64.6 (56.4, 70.7)
Median tumor size in mm	40.0 (24.0, 80.0)
Number of tumors	
1	301 (63%)
≥2	175 (37%)
Gender	
Male	436 (78%)
Female	125 (22%)
Etiology of liver disease	
Alcohol abuse	172 (31%)
HCV	114 (20%)
HBV	106 (19%)
NASH	42 (7.5%)
Hemochromatosis	25 (4.5%)
Unknown/Other	131 (23%)
BCLC	
A	104 (19%)
B	356 (63%)
C	63 (11%)
D	38 (6.8%)
ECOG PST	
0–1	533 (96%)
2	12 (2.2%)
3	6 (1.1%)
4	7 (1.3%)
Liver cirrhosis	
Absent	196 (35%)
Present	365 (65%)
Child–Pugh score	
A	208 (53%)
B	144 (37%)
C	38 (9.7%)
Portal vein thrombosis (PVT)	65 (12%)
Vascular invasion	
Absent	369 (66%)
Micro	110 (20%)
Macro	82 (15%)

[1] Statistics presented: median (IQR); *n* (%).

Table S2. Clinicopathologic data associated with high and low combined CAD-CPS1 score.

Characteristic	CAD–CPS1 Low	CAD–CPS1 High	*p*-Value [2]
	N = 359 (68%) [1]	*N* = 171 (32%) [1]	
Median age in years (range)	65.0 (56.7, 71.1)	63.1 (56.2, 70.7)	0.2
Median tumor size in mm	39.0 (24.0, 74.5)	45.0 (29.5, 93.0)	0.019
Number of tumors			0.6
1	191 (62%)	96 (65%)	
≥2	116 (38%)	51 (35%)	
Gender			0.005

Male	296 (82%)	122 (71%)	
Female	63 (18%)	49 (29%)	
Etiology of liver disease			
Alcohol abuse	122 (34%)	36 (21%)	0.003
HCV	77 (21%)	29 (17%)	0.3
HBV	63 (18%)	35 (20%)	0.5
NASH	26 (7.2%)	15 (8.8%)	0.7
Hemochromatosis	16 (4.5%)	8 (4.7%)	>0.9
Unknown/Other	80 (22%)	46 (27%)	0.3
BCLC			<0.001
A	76 (21%)	21 (12%)	
B	216 (60%)	122 (71%)	
C	36 (10%)	25 (15%)	
D	31 (8.6%)	3 (1.8%)	
ECOG PST			0.6
0–1	343 (96%)	164 (98%)	
2	8 (2.2%)	1 (0.6%)	
3	4 (1.1%)	1 (0.6%)	
4	4 (1.1%)	2 (1.2%)	
Liver cirrhosis			0.001
Absent	112 (31%)	79 (46%)	
Present	247 (69%)	92 (54%)	
Child–Pugh score			0.03
A	132 (50%)	59 (58%)	
B	99 (38%)	39 (39%)	
C	31 (12%)	3 (3.0%)	
Portal vein thrombosis	37 (10%)	27 (16%)	0.1
Vascular invasion			<0.001
Absent	259 (72%)	83 (49%)	
Micro	53 (15%)	56 (33%)	
Macro	47 (13%)	32 (19%)	
Tumor grading			<0.001
G1	81 (25%)	18 (12%)	
G2	187 (58%)	76 (49%)	
G3	52 (16%)	62 (40%)	
Macrotrabecular subtype			<0.001
no	341 (97%)	141 (82%)	
yes	10 (2.8%)	30 (18%)	
VETC			0.2
negative	300 (84%)	135 (79%)	
positive	59 (16%)	36 (21%)	
GS overexpression			0.008
no	273 (76%)	147 (86%)	
yes (≥9)	86 (24%)	23 (14%)	

[1] Statistics presented: median (IQR); *n* (%). [2] Statistical tests performed: Wilcoxon rank-sum test; chi-square test of independence; Fisher's exact test.

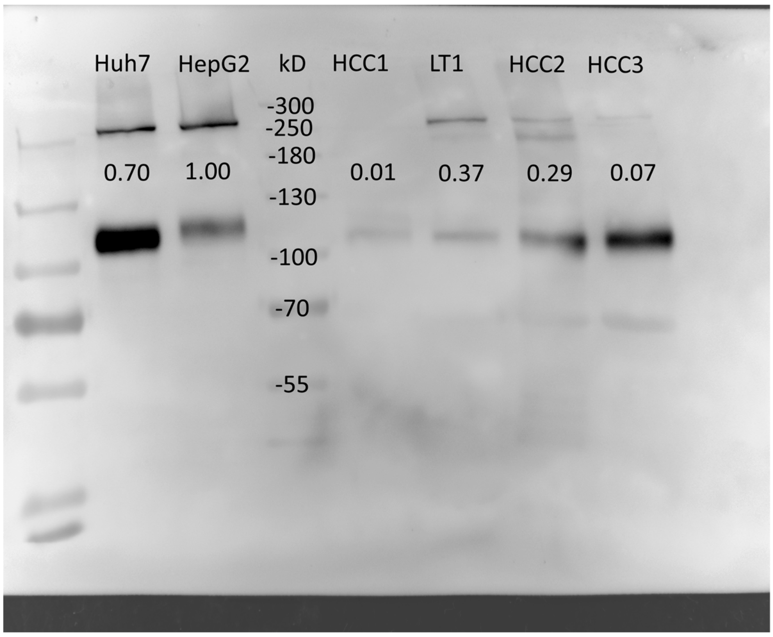
Huh7
HepG2
kD
HCC1
LT1
HCC2
HCC3
-300
-250
-180
-130
-100
-70
-55
0.70
1.00
0.01
0.37
0.29
0.07

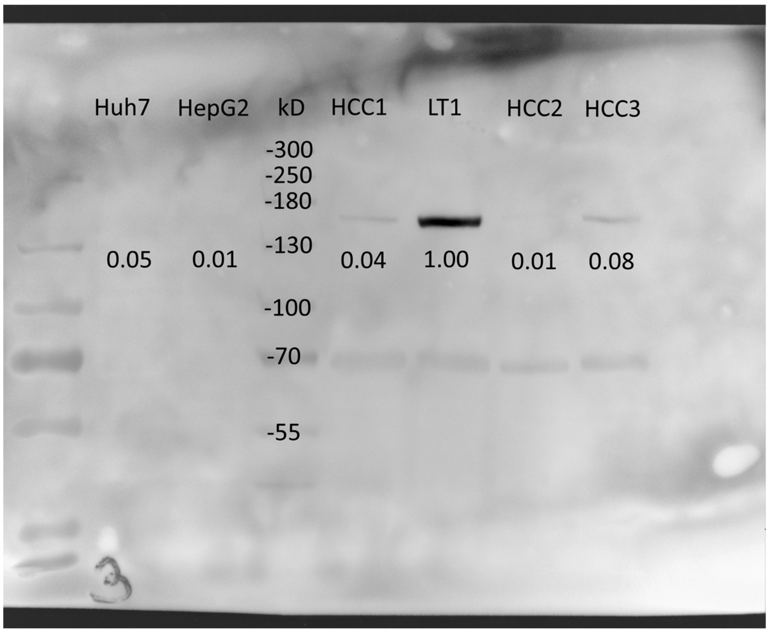
Huh7
HepG2
kD
HCC1
LT1
HCC2
HCC3
-300
-250
-180
-130
-100
-70
-55
0.05
0.01
0.04
1.00
0.01
0.08

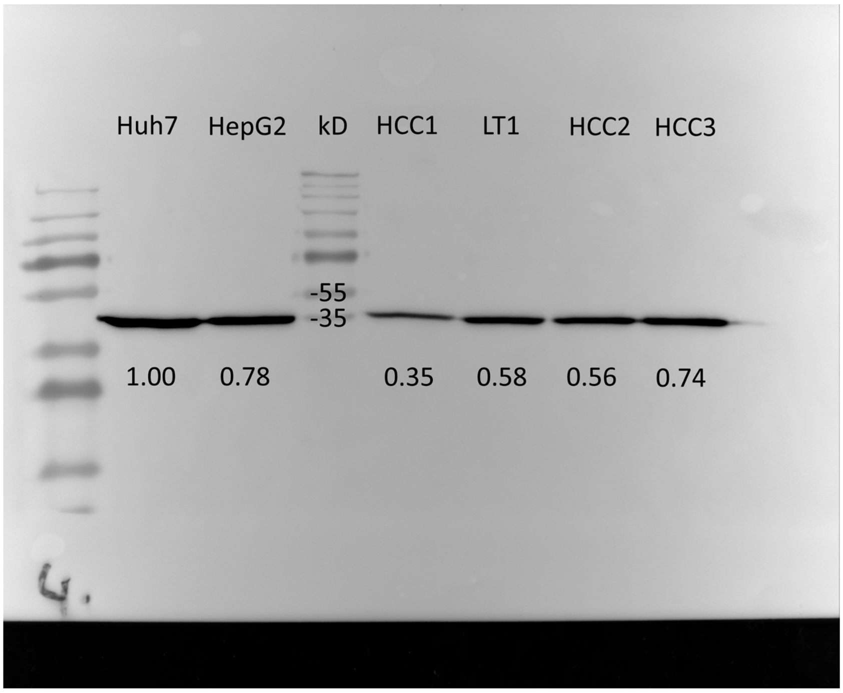

Figure S5. Original images of the western blots shown in Figure S1.Densitometric readings are displayed below the band of interest.
